# Uridine diphosphate glucuronosyl transferase 1A *(UGT1A1)* promoter polymorphism in young patients with sickle cell anaemia: report of the first cohort study from Nigeria

**DOI:** 10.1186/s12881-019-0899-3

**Published:** 2019-10-16

**Authors:** Oladele Simeon Olatunya, Dulcineia Martins Albuquerque, Ganiyu Olusola Akanbi, Olufunso Simisola Aduayi, Adekunle Bamidele Taiwo, Opeyemi Ayodeji Faboya, Tolorunju Segun Kayode, Daniela Pinheiro Leonardo, Adekunle Adekile, Fernando Ferreira Costa

**Affiliations:** 1 Hematology and Hemotherapy Center (Hemocentro), University of Campinas (UNICAMP), Rua Carlos Chagas, 480, Barão Geraldo, Campinas, SP 13083-970 Brazil; 2 Department of Paediatrics, College of Medicine, Ekiti State University, Ado Ekiti, Ekiti State Nigeria; 3 Department of Radiology, College of Medicine, Ekiti State University, Ado Ekiti, Nigeria; 4 Department of Paediatrics, Ekiti State University Teaching Hospital, Ado Ekiti, Nigeria; 5 Department of Medical Biochemistry, College of Medicine, Ekiti State University, Ado Ekiti, Nigeria; 6 Department of Chemical Pathology, Ekiti State University Teaching Hospital, Ado Ekiti, Nigeria; 7 Department of Pediatrics, Faculty of Medicine, Kuwait University, Jabriya, Kuwait

**Keywords:** Sickle cell anaemia, Laboratory parameters, Clinical events, Gallstone, *UGT1A1* polymorphism, Nigeria

## Abstract

**Background:**

(TA) n repeat sequence (rs8175347) of *UGT1A1* gene promoter polymorphism is associated with serum bilirubin levels and gallstones among different sickle cell anaemia (SCA) populations. There are no data on *UGT1A1* polymorphisms and their impact on Nigerian SCA patients. In this study, we determined the distribution of the *UGT1A1* (TA) n genotypes among a group of young Nigerian SCA patients and healthy controls. In addition, the influence of *UGT1A1* (TA) n genotypes on the laboratory and clinical events among the patients was determined.

**Methods:**

The distribution of the *UGT1A1* (TA) n genotypes among 101 young Nigerian SCA patients and 64 normal appropriate controls were determined and studied. The *UGT1A1* (TA) n genotypes were further classified into subgroups and used to differentiate the clinical events and laboratory parameters of the patients.

**Results:**

Four (TA) n alleles:(TA)5, 6, 7, and 8 were found. These were associated with 10 genotypes: TA5/5, 5/6, 5/7, 5/8, 6/6, 6/7, 6/8, 7/7, 7/8, 8/8. The normal (wild-type)-(TA) 6/6), low- (TA) 7/7, 7/8, 8/8), intermediate- (TA) 5/7, 5/8, 6/7, 6/8), and high-activity (TA) 5/5, 5/6,) genotypes were found in 24.8, 24.8, 41.5, and 8.9% patients and 20.3, 15.6, 61, and 3.1% controls respectively. The general genotype distribution of the patients and control group were not significantly different. There were significant differences in serum bilirubin and lactate dehydrogenase (LDH) of the patients when differentiated by the *UGT1A1* (TA) n genotypes (*p*<0.05). Asymptomatic gallstones were found in 5.9% of patients and were significantly of the low-activity genotypes sub-group 5 (20%) vs 1(1.3%) *p* = 0.0033. Although, bilirubin and fetal hemoglobin (HbF) of patients with gallstones were significantly different from those without gallstone, only the serum bilirubin was associated with *UGT1A1* (TA) n genotypes on multivariate analysis (*p* < 0.0001).

**Conclusion:**

This study highlights the contribution of *UGT1A1* polymorphisms, a non-globin genetic factor, to the laboratory and clinical manifestations of young Nigerian SCA patients for the first time. It also shows that children with co-inheritance of low *UGT1A1* (TA) n affinity genotypes may be at risk of gallstone, hence the need to follow them up.

## Introduction

Sickle cell disease (SCD) is a common genetic disorder among Africans. Individuals with the disease have variable clinical expression but homozygosity for the HbS gene, also known as sickle cell anaemia (SCA), is the most severe form [1]. Children with SCA have chronic hemolysis, leading to accumulation of serum bilirubin and consequent gallstones [2]. Bilirubin is a tetrapyrol that results from the breakdown of heme in red blood cells. At moderate levels, it protects against oxidative stress and inflammatory injuries, and some infectious diseases [3–5]. However, excessive bilirubin levels, as seen in chronic hemolysis, have been linked to increased incidence of gallstones [1, 2, 5]. In children with SCA, this risk increases with advancing age with a cumulative incidence of approximately 50% by adulthood and some of them may need cholecystectomy [2, 6, 7].

Uridine diphosphate glucuronosyltransferase 1A isoform 1 *(UGT1A1)* is a member of the superfamily of phase II conjugating enzymes that aids the elimination of bilirubin, drugs and a vast variety of endogenous and exogenous substrates by adding a glucuronide moiety to the substrates [8, 9]. Genetic mutations resulting in absence or severely reduced *UGT1A1* activity leads to Criggler-Najjar syndrome which is characterized by severely elevated serum bilirubin and increased risk of kernicterus [10]. However, variations in (TA) n tandem repeat sequence within the TATA box promoter region affect *UGT1A1* gene expression and the activity of its (TA) n four alleles, namely; (TA) 5, 6, 7, and 8, leading to moderate elevation of serum bilirubin [8, 9]. There is a negative association between the *UGT1A1* and repeat length of the four alleles attributable to the decreasing promoter activity acting via altered affinity for the TATA–binding protein [8, 9]. The promoter activity leads to functional changes such that, genotype (TA)5/5 is considered to have ~ 20% increased expression i.e. high activity in comparison to genotype (TA) 6/6 known as the wild-type with normal activity. In addition, genotypes heterozygous and or homozygous for (TA)7 and (TA)8 alleles have approximately between 30 and 50% reduced expression i.e. low activity, compared to the wild-type [8]. However, genotypes (TA)7/7, 7/8 & 8/8 have the lowest activity [2, 8, 9] and (TA)7/7 has been described generally as genetic hallmark for Gilbert syndrome [2, 11]. There is an inverse relationship between the serum bilirubin levels across these subgroups and the degree of genotype activity. To this end, individuals with low-activity genotypes have elevated levels of serum bilirubin and are, therefore, subjected to the modulating effects of higher serum bilirubin levels including susceptibility to gallstones [2, 8, 9, 11].

Despite the huge burden of SCA in Africa [1], with Nigeria having the highest burden of SCA in the world [12], there is little understanding of the contributions of genetic modifiers of SCA phenotypes in the country. To the best of our knowledge, there are no data on the effects of *UGT1A1* polymorphisms on the clinical expression of Nigerian SCA patients. The aims of this study were to determine the distribution of *UGT1A1* (TA) n genotypes among a group of young SCA patients and healthy controls and also determine the influence of the *UGT1A1* (TA) n genotypes on the laboratory parameters and clinical events among the young SCA patients.

## Methods

### Study participants and settings

The study was conducted on 101 hydroxyurea-naïve children and adolescents with SCA (Homozygous SS) aged between 2 and 21 years (median of 9 years) who are regular attendees at the paediatric haematology unit of the Ekiti State University Teaching Hospital (EKSUTH), Ado Ekiti, Ekiti State, in Southwest Nigeria. They were all in steady state at the time of recruitment. Steady state was defined as being free from any acute event(s) for at least 1 month and transfusion free for at least 4 months. Sixty four genetically independent and unrelated children, who accompanied their siblings to or attended the paediatrics outpatients’ well-child clinic of the hospital served as the controls. Participants with confirmed or suspected liver or other chronic diseases apart from SCA were excluded. Also excluded were the few SCA patients on regular blood transfusion and/or hydroxyurea.

### Ethical approval

The study was approved by the Ethics and Research Committee of EKSUTH no: A67/2016/03/003. Because the DNA analysis was done at the hematology and hemotherapy centre, University of Campinas, the study was also approved by the University of Campinas Ethics Committee no: CAAE 54031115.9.0000.5404. Written informed consent of parents/caregivers as well as patients’ assents and consents were obtained as applicable after explaining the purpose of the study to them in clear and plain language.

### Data collection

#### Clinical and laboratory data

Information regarding the steady-state laboratory parameters and clinical events of the patients were retrieved from their hospital records. Average of at least two steady state results of laboratory parameters performed between 3 to 6 months intervals by standard techniques were recorded for each participants. The steady state parameters included the complete blood performed by Sysmex KX21N Hematology analyser (Sysmex Corporation, Kobe, Japan). The serum lactate dehydrogenase (LDH), bilirubin, were measured with standard techniques. The quantitative assessment of haemoglobin pattern (HbF and HbS), was done by high performance liquid chromatography (HPLC, Bio-Rad Variant D10, USA).

Other information retrieved from patients’ charts included the biodata and details of the clinical evolution of SCA such as number of vaso-occlussive crisis (VOC) i.e. severe bone pain crises that disrupt daily activities and required admission and/or administration of opioids within the preceding 1 year, leg ulcer, priapism, overt osteonecrosis and/or overt stroke as well as presence of gallstone as determined by serial abdominal ultrasound scans conducted on the patients as clinically indicated. In addition, the clinical records of the patients with gallstones were examined for the presence or absence of symptoms, and or treatment(s) for gallstone complications. The definitions of clinical events were as previously described [13].

#### Genetic studies

These were carried out at the Centro de Hematologia e Hemoterapia (Hemocentro), UNICAMP, Campinas, São Paulo State, Brazil. SCA was initially diagnosed by haemoglobin electrophoresis and high performance liquid chromatography (HPLC) in Nigeria.

The genomic DNA of each participant was extracted from peripheral blood leukocytes by Qiagen QIAamp DNA Blood Mini Kit, (Cat No. 51104 Germany), according to the manufacturer’s protocols and used to confirm the molecular diagnosis of SCD by polymerase chain reaction (PCR). The DNA purity and concentration were evaluated on Nanodrop ND-1000™ Spectrophotometer (NanoDrop Technologies, Inc., DE, USA). PCR for exon 1 of HBB gene amplification was performed according to the following protocol in 30 μL volume: 150 ng of genomic DNA; 1X Colorless Go*Taq*® Flexi Reaction Buffer (Promega Corporation, Madison, USA); 2 mM MgCl_2_; 0.2 mM of dNTP mix; 0.2 μM of each primer (Integrated DNA Technologies, Coralville, Iowa) named P1: TCCTAAGCCAGTGCCAGAAG and P5: TCATTCGTCTGTTTCCCATTC [14] and 1 U of Go*Taq®* Flexi DNA Polymerase (Promega Corporation, Madison, USA). Thermal cycle conditions were as follow: preheating at 96 °C for 2 min, and then 35 cycles of amplification, with 30 s at 96 °C for denaturation, 30 s at 58 °C for annealing, and 60 s at 72 °C for elongation. The PCR product (771 bp) was submitted to sequencing reaction by the following conditions: 30 ng PCR product, 1,0 μL BigDye Terminator v3.1 Ready Reaction Mix (AppliedBiosystems, Foster City, CA, USA), 1X BigDye Reaction Buffer, 2 μM of primer (P1 or P5). After thermal cycling (preheating at 96 °C for 2 min, followed by 25 cycles of 96 °C for 15 s, 58 °C for 5 s, 60 °C for 4 min), the sequencing reaction product was precipitated by ammonium acetate and ethanol method, dried at 65 °C and re-suspended in 10 μL of Hi-Di Formamide for electrophoresis. Amplified fragments were separated by capillary electrophoresis on an ABI3500 Genetic Analyzer (Applied Biosystems, CA).

The UGT1A1 rs8175347 SNP identification was performed by Polymerase Chain Reaction (PCR) as previously described [15, 16], with some modifications. Briefly, the PCR reaction was prepared in 30 μL volume with 100 ng of genomic DNA; 1X Reaction Buffer (BIOTOOLS B&M Labs, Spain); 2.16 mM MgCl_2_; 1.33 mM of dNTP mix; 133 nM of each primer: UGT1A1_*F: 5′-FAM /GTCACGTGACACAGTCAAAC − 3′ (*labelled with fluorescein amidite – 6-FAM) and UGT1A1_R: 5′ CAACAGTATCTTCCCAGCATG − 3′) (Integrated DNA Technologies, Coralville, Iowa), and 1 U *Taq* DNA Polymerase (BIOTOOLS B&M Labs, Spain). Thermal cycle conditions were as follow: preheating at 96 °C by 2 min, followed by 25 cycles of 96 °C for 30 s, 58 °C for 40 s, and 72 °C for 40 s. An ended step at 72 °C for 30 min was performed to promote adenylation of the PCR products. Fragment analysis was performed by Capillary electrophoresis on ABI3500 Genetic Analyzer and sizes of amplicons were calculated by Gene Mapper *v*4.1 Software (both Applied Biosystems, Carlsbad, CA) - the fragments ranged from 197 to 203 bp, corresponding to (TA)_5_ - (TA)_8_ repeats (Additional file 1: Figure S1). Based on literature data, the *UGT1A1* (TA) n genotypes were further classified into four subgroups namely: Wild-type (normal), low-, intermediate-, and high-activity subgroups as previously described [2, 8, 9, 11]. All DNA studies were carried out blinded regardless of the clinical and laboratory parameters of the participants.

#### Data analysis

Statistical analysis was performed with the GraphPad Prism Program, version 5 for Windows (San Diego, California, USA). The normal distribution of the quantitative variables was verified by the Kolmogorov-Smirnov and Shapiro-Wilk tests. The frequencies of variables were described and the significance of differences between quantitative variables across groups of patients was assessed using the Kruskal-Wallis analysis of variance (ANOVA) for ≥3groups, and Mann-Whitney test for two groups. Chi-square or Fisher’s exact tests were used as appropriate for the categorical variables. Odd ratios were obtained by applying logistic regression to determine the effects of *UGT1A1* promoter polymorphism using the *UGT1A1* (TA) n genotype as the independent variable and other outcomes of interest as the dependent variables. The test for Hardy-Weinberg equilibrium was performed using the R-Project for statistical computing web tool available at https://www.R-project.org. Level of significance was set at *P* < 0.05 for all statistical analyses.

## Results

The 101 patients with SCA consisted of 66 males and 35 females with median age of 9 and a range 2 - 21 years. The controls were made up of 19 sickle cell trait (HbAS) and 45 haemoglobin HbAA, median age of 8, range 2 - 18 years (*p* = 0.4260), and 41 males. The SCA patients have been on follow up for a median of 4 years, range 1–14 years.

### *UGT1A1* (TA) n alleles and genotypes

Four (TA) n alleles: (TA)5, 6, 7, and 8 were found with gene frequencies of 0.11, 0.43, 0.41 and 0.05 respectively. The alleles were associated with 10 genotypes: TA5/5, 5/6, 5/7, 5/8, 6/6, 6/7, 6/8, 7/7, 7/8, 8/8 (Table 1). The wild-type (normal) (TA) 6/6), low (TA) 7/7, 7/8, 8/8), intermediate (TA) 5/7,5/8, 6/7, 6/8), and high (TA) 5/5, 5/6), enzyme activity genotypes were found in 24.8, 24.8, 41.5%, & 8.9% patients and 20.3, 15.6, 61%, & 3.1% controls respectively. The low activity genotypes were found in 25 (24.7%) patients and 10 (15.6%) of the controls (*P* = 0.1773). Homozygous (TA) n TA7/7 was found in 22 (21.7%) patients and 5 (7.8%) controls *p* = 0.018 (Table 1). The observed genotype distributions of the patients and control group were not significantly different from the values expected under Hardy-Weinberg equilibrium (*x*^2^ = 15.10, df = 9, *p* = 0.09), and (*x*^2^ = 11.86, df = 9, *p* = 0.22), respectively.

**Table 1:** Allele and genotype frequencies of UGT1A1 promoter polymorphisms among participants

Variables	SCA (*N* = 101)	AS (*N* = 19)	AA (*N* = 45)
	Freq n (%)	Freq n (%)	Freq n (%)
Allelotypes			
(TA) 5	18 (11.7)	2 (6.7)	10 (12.5)
(TA)6	67 (43.5)	16 (53.3)	28 (35.0)
(TA)7	61 (39.6)	10 (33.3)	37 (46.2)
(TA)8	8 (5.2)	2 (6.7)	5 (6.3)
UGT1A1 Genotypes			
TA5/5	0 (0)	0 (0)	1 (2.2)
TA5/6	9 (8.9)	0 (0)	1 (2.2)
TA5/7	6 (6.0)	1 (5.2)	8 (17.7)
TA5/8	3 (2.9)	0 (0)	0 (0)
TA6/6	25 (24.7)	8 (42.1)	5 (11.1)
TA6/7	31 (30.7)	7 (36.8)	21 (46.7)
TA6/8	2 (2.0)	1 (5.2)	1 (2.2)
TA7/7	22 (21.7)	1 (5.2)	4 (8.9)
TA7/8	2 (2.0)	1 (5.2)	4 (8.9)
TA8/8	1 (1.0)	0 (0)	0 (0)
UGT1A1 Genotypes by degree of Activity			
Low-Activity genotypes TA (7/7, 7/8, 8/8)	25 (24.8)	2 (10.5)	8 (17.8)
Intermediate-Activity genotypes (TA6/7, TA6/8), TA5/7, TA5/8,	42 (41.5)	9 (47.4)	30 (66.7)
Normal Activity genotypes i.e. (Wild Type) TA6/6	25 (24.8)	8 (42.1)	5 (11.1)
High-Activity genotypes TA5/5, TA5/6,	9 (8.9)	0 (0)	2 (4.4)

### Effects of *UGT1A1* genotype on serum bilirubin and other laboratory parameters of patients

Both the total bilirubin and unconjugated bilirubin levels showed distinct quantitative patterns across the *UGT1A1* (TA) n genotype subgroups with the low-activity genotype group having the highest levels of serum bilirubin (*p* < 0.0001). The LDH also showed a similar pattern (*p* = 0.0002). However, this was not demonstrated in the other laboratory parameters (Table 2). The stratification of the individual *UGT1A1* (TA) n genotypes separately shows that (TA)7 and (TA) 8 alleles were associated with higher levels of both serum bilirubin and LDH in general (Additional file 2: Table S1).

**Table 2:** Influence of *UGT1A1* (TA) n genotype on laboratory parameters of patients

Parameter	a. Low activity UGT1A1 genotypes *N* = 25	b. Intermediate activity UGT1A1 genotypes *N* = 42	c. Normal activity UGT1A1 genotypes (i.e Wild type) *N* = 25	d. High activity UGT1A1 genotypes *N* = 9	Anova (Kruskal-Wallis Test) *P* values (a vs b vs c vs d)
Biochemical and haematologic	Median (Range)	Median (Range)	Median (Range)	Median (Range)	
Total Bilirubin (mg/dl)	2.8 (1.2–8.1)	1.8 (0.8–4.6)	1.4 (0.4–3.8)	1.4 (0.5–2.8)	< 0.0001** ^**1**^
Unconjugated Bilirubin (mg/dl)	1.8 (0.6–6.3)	0.8 (0.1–3.3)	0.6 (0.1–2.3)	0.5 (0.3–1.6)	< 0.0001** ^**1**^
LDH (IU/L)	987 (296–1860)	798 (215–1489)	789 (340–1417)	287 (197–800)	0.0002** ^**2**^
AST (IU/L)	46 (18–89)	37 (8–89)	42 (7–89)	39 (18–89)	0.4837**
ALT (IU/L)	25 (4–65)	19 (7–77)	20 (7–44)	12 (4–34)	0.216**
Hb conc (g/dl)	7.3 (6.3–10)	7.5 (6.3–9.7)	7.2 (6.2–10)	7.9 (7–8.8)	0.608**
MCV (fl)	80.6 (66.9–104.1)	82.3 (60.3–10.2)	77.3 (63.9–96.3)	81 (55.9–115)	0.642**
RBC (× 10^12^/L)	2.7 (1.9–4.1)	2.7 (1.8–4.1)	2.9 (1.8–4.8)	2.8 (2.2–3.9)	0.258**
WBC (× 10^9^/L)	13 (8.5–26)	13.4 (6.1–29.3)	13.3 (7–25)	12.2 (7.6–23.1)	0.889**
Platelet (× 10^9^/L)	367 (118–771)	349 (159–601)	361 (108–669)	391 (135–832)	0.955**
HbF (%)	9.7 (1.3–20.6)	8.2 (1.7–24.4)	10.7 (2.5–32)	9.4 (0.9–28.5)	0.86**
HbS (%)	80 (71–91.5)	82 (44–91.5)	80 (44–88.3)	80 (65–89)	0.90**
HbA2 (%)	1.6 (0.5–3.5)	1.7 (0.2–3.8)	1.5 (0.2–4.0)	1.1 (0.3–3.1)	0.3147**

Significant *p* values are indicated in bold fonts

*HbF* Fetal hemoglobin, *RBC* Red blood cell, *Hb* Hemoglobin concentration, *HbS* Hemoglobin S, *HbA2* Hemoglobin A2, *MCV* Mean corpuscular volume, *WBC* White blood cell count, *LDH* Lactate dehydrogenase, *AST* Aspartate transaminase, *ALT* Alanine transaminase

** = Kruskal-Wallis Test with Dunn’s multiple comparison post-hoc tests with differences in *1 = (a vs b), (a vs c), (a vs d) only; *2 = (a vs d), (b vs d), (c vs d) only

### Effects of *UGT1A1* (TA) n genotype on clinical events

Asymptomatic gallstones were found in 6 (5.9%) patients. Gallstones were significantly more common in patients with low-activity genotypes compared to all the other remaining genotype subgroups 5 (20%) vs 1(1.3%) *p* = 0.0033, (Table 3). These were 2 females and 4 males; the two females were aged 10 and 13 years respectively, while the males were aged 10, 13, 15 and 16 years respectively. Four of the patients with gallstone had TA 7/7 genotypes, the remaining two each had TA 7/8, or TA 6/7. No significant relationship was found with the other clinical events.

**Table 3:** Influence of *UGT1A1* (TA) n genotype on clinical events of patients

Clinical events	a. Low activity UGT1A1 genotypes *N* = 25	b. Intermediate activity UGT1A1 genotypes *N* = 42	c. Normal activity UGT1A1 genotypes (Wild Type) *N* = 25	d. High activity UGT1A1 genotypes *N* = 9	*P* value a vs (b + c + d)
VOC rate per year	2 (0–6)	1.5 (0–6)	1 (0–6)	0 (0–6)	0.2218*
Overt Stroke	1	2	1	0	1.000†
No overt stroke	24	40	24	9	
Osteonecrosis	1	2	2	0	1.000†
No osteonecrosis	24	40	23	9	
Leg ulcer	0	2	4	0	0.331†
No Leg ulcer	25	40	21	9	
Gallstones	5	1	0	0	**0.0033†**
No Gallstone	20	41	25	9	
Priapism (Male only event, *N* = 66)					
Priapism	2	3	0	0	0.594†
No Priapism	15	25	16	5	

Significant *p* values are indicated in bold fonts

*VOC* Vaso-occlussive crisis

* = Mann-Whitney Test, † = Fisher’s exact test

### Comparison of laboratory parameters between patients with and without gallstones

There were significant differences between the serum bilirubin and HbF levels in patients with gallstones when compared with those without. No difference was observed in the LDH and age of the two groups. Furthermore, when those with gallstones were compared with age- and sex-matched patients within the same *UGT1A1* (TA) n genotype subgroup, only serum bilirubin and HbF showed significant differences between the two groups (Table 4).

**Table 4:** Comparison of parameters in patients with and without gallstones

Parameter	Patients with gallstones (*N* = 6) Median (Range)	Patients without gallstones (*N* = 95) Median (Range)	*P* value
Total Bilirubin (mg/dl)	6.4 (2.8–8.1)	1.8 (0.4–6.7)	0.0001*
Unconjugated Bilirubin (mg/dl)	4.7 (0.9–6.3)	0.79 (0.1–5)	0.0007*
LDH (IU/L)	1004 (592–1860)	794 (197–1750)	0.1263*
HbF (%)	4.7 (1.3–6.8)	10.2 (0.9–32)	0.0107*
Hb (g/dl)	7.1 (6.3–8.8)	7.5 (6.2–10)	0.4210*
Age in years	11.5 (8–16)	9 (2–21)	0.1368*
Sex			
Male (*n* = 66)	4	62	1.000†
Female (*n* = 35)	2	33	
Parameter	Patients with gallstones (*N* = 6) Median (Range)	Matched peers without gallstones within same UGT1A1 genotype activity group *N* = 10 Median (Range)	*P* value
Total Bilirubin (mg/dl)	6.4 (2.8–8.1)	2.2 (1.9–3.2)	0.0023*
Unconjugated Bilirubin (mg/dl)	4.7 (0.9–6.3)	1.2 (1.0–2.0)	0.0020*
LDH (IU/L)	1004 (592–1860)	890 (340–1603)	0.628*
HbF (%)	4.7 (1.3–6.8)	14.7 (4.2–17.9)	0.022*
Hb (g/dl)	7.1 (6.3–8.8)	8.0 (6.5–8.9)	0.137*

NB Significant *P* values are indicated in bold fonts

*HbF* Fetal hemoglobin, *Hb* Hemoglobin concentration, *LDH* Lactate dehydrogenase

* = Mann-Whitney test, † = Fisher’s Exact test

### Relationship between *UGT1A1* (TA) n genotypes and other parameters by multivariate analysis

Unconjugated bilirubin was significantly associated with the low activity *UGT1A1* (TA) n genotypes (Adjusted Odd Ratio (1.08), 95% Confidence interval (1.034768–1.127873), *P* < 0.0001). Also, significant association was found with the total bilirubin when it was used in place of unconjugated bilirubin in the logistic regression model (Adjusted Odd Ratio (1.05), 95% Confidence interval (1.029172–1.089832), *P* < 0.0001). No association was found with the other laboratory parameters.

## Discussion

There are gaps in the understanding of the impacts of genetic modifiers on SCA phenotype among African patients. Given the distinct segregation of genetic markers among different populations, it is pertinent that more studies are carried out among diverse ethnic cohorts to fully understand the impact of genetic polymorphisms in SCA.

This study confirms the variability of bilirubin levels based on the activity of the *UGT1A1* (TA) n genotypes as previously reported [2, 4, 5, 8, 9]. However, we are not aware of any previous study that has described the stratification of LDH among SCA patients based on *UGT1A1* (TA) n genotype activity as found in this study. While the *UGT1A1* modulation of serum bilirubin levels is well understood [2, 17, 18], the exact mechanism through which *UGT1A1* could be associated with LDH is not clear. However, it should be noted that they are both markers of hemolysis [19]. Given the association of LDH with some phenotypes of SCA [19, 20], there is need to further unravel the link between LDH and *UGT1A1* activity.

Gilbert syndrome (GBS) has been described in individuals with the TA7/7 genotype [2, 4, 11, 17]. The proportion of patients with TA7/7 genotype in this study (21.7%) is higher than between 3 and 18% described among Europeans [21, 22] and Brazilians of different descents [23–25]. Similarly, it is higher than the 6% found among Kuwaiti SCA patients [26] and the 5 to 11% among other Africans [11, 22]. However, it is lower than the 32% described among the SCA patients in the USA [27]. Nevertheless, the TA7/7 genotype prevalence in this study, is comparable to between 18.2 and 20.3% earlier described among Nigerians with non-SCD related illnesses [28, 29]. However, the higher preponderance of TA7/7 among the patients compared to the controls in the present study is not clear but may be due to the low sample size.

Besides the TA7/7 genotype, the other *UGT1A1* (TA) n genotypes found in this study have been described among Africans [9, 22]. These observations indicate that the *UGT1A1* (TA) n genotype is quite variable among Nigerians. It also confirms the suggestions that the expression of the *UGT1A1* (TA) n genotype variants is heterogeneous among Africans compared to Caucasians [9, 21, 22].

Our finding that the low-activity *UGT1A1* (TA) n genotype was associated with gallstones confirms previous observations that SCA patients with the low-activity *UGT1A1* (TA) n genotypes especially the TA7/7, are at risk of developing gallstones [2, 6, 7, 11, 17]. In addition to the TA7/7 genotype, other authors [11, 30], have reported that some other low-activity *UGT1A1* (TA) n genotypes like TA7/8 and TA8/8 predispose SCA patients to gallstones as found in this study.

The proportion of patients with asymptomatic gallstones in this study (5.9%), is comparable to between 4 and 6% earlier reported among Nigerian children of similar age to our SCA cohorts [31–33]. This is also similar to the 4% found in Ghana [34], a close neighbour to Nigeria. However, it is lower than between 9 and 58% reported for some other African [35–37], Italian [38], American [39], and Brazilian [40] patients. These observations possibly highlight the variations in propensity to gallstone development among children with SCA from different backgrounds.

Despite the observation that the *UGT1A1* (TA) n low -activity genotype is a leading factor in hyperbilirubinemia and lithogenesis among SCA patients [2, 6, 7, 41, 42], the impact of *UGT1A1* polymorphism on the phenotypic expression of the Nigerian SCA patients was unknown prior to this study as none of the previous studies from Nigeria examined the *UGT1A1* of the patients [31–33]. There is therefore, the need to closely follow up these patients given that, results of follow-up studies have indicated higher prevalence of gallstones and its complications with increasing age of SCA patients [2, 26, 30, 37, 40].

Beyond bilirubin metabolism and gallstone development, it has been speculated that individuals with the low-activity genotypes may be subjected to some other modulating effects of higher serum bilirubin levels that are often associated with these genotypes [2, 8, 9, 11]. These other modulating effects include protection against oxidative stress, inflammatory injuries, and reduced susceptibility to infections [2–5, 8, 9, 43]. We did not observe any association between the *UGT1A1* (TA) n genotypes and any of the other SCA downstream events/phenotypes examined (VOC, leg ulcer, priapism, overt stroke and osteonecrosis) that could be perturbed by both inflammatory and oxidative injuries in SCA. However, the small sample size of the study makes it difficult to draw any firm conclusion.

The major limitations of the present study are its hospital-based nature and the small sample. Despite these, it was able to confirm that *UGT1A1* (TA) n genotypes are tightly associated with bilirubin and LDH levels, and the development of gallstones among young Nigerians with SCA. In addition, it also suggests that the pathway to elevated serum bilirubin and gallstone development, among our study cohort, may not be exclusively driven by hemolysis but also by *UGT1A1* polymorphisms.

## Conclusions

This study shows that children with co-inheritance of low *UGT1A1* (TA) n affinity genotypes may be at risk of gallstone, thus highlighting the need to closely follow them up for early identification of possible gallstone complications and provision of appropriate intervention(s). In addition, it highlights the contribution of *UGT1A1* polymorphisms, a non-globin genetic factor, to the laboratory and clinical manifestations of young Nigerian SCA patients for the first time.

## Supplementary information

**Additional file 1: Figure S1.** Supplement material for *UGT1A1* polymorphism in young Nigerians with SCA.

**Additional file 2: Table S1.** *UGT1A1* genotypes distribution with their bilirubin and LDH levels among patients.

## Data Availability

The datasets generated and or analysed during the current study are available from the corresponding author on a reasonable request.

## Abbreviations

EKSUTH: Ekiti State University Teaching Hospital
GBS: Gilbert syndrome
HbF: Fetal hemoglobin
HPLC: High performance liquid chromatography
LDH: Lactate dehydrogenase
PCR: Polymerase chain reaction
SCA: Sickle cell anaemia
SCD: Sickle cell disease
UGT1A1: Uridine Diphosphate Glucuronosyl Transferase 1A
UNICAMP: University of Campinas
VOC: Vaso-occlussive crisis
