# Supplementary figures and images for: Uridine diphosphate glucuronosyl transferase 1A (UGT1A1) promoter polymorphism in young patients with sickle cell anaemia: report of the first cohort study from Nigeria
Source: BMC Med Genet. 2019 Oct 16;20:160. doi: 10.1186/s12881-019-0899-3 (PMC6794735; doi:10.1186/s12881-019-0899-3)

**
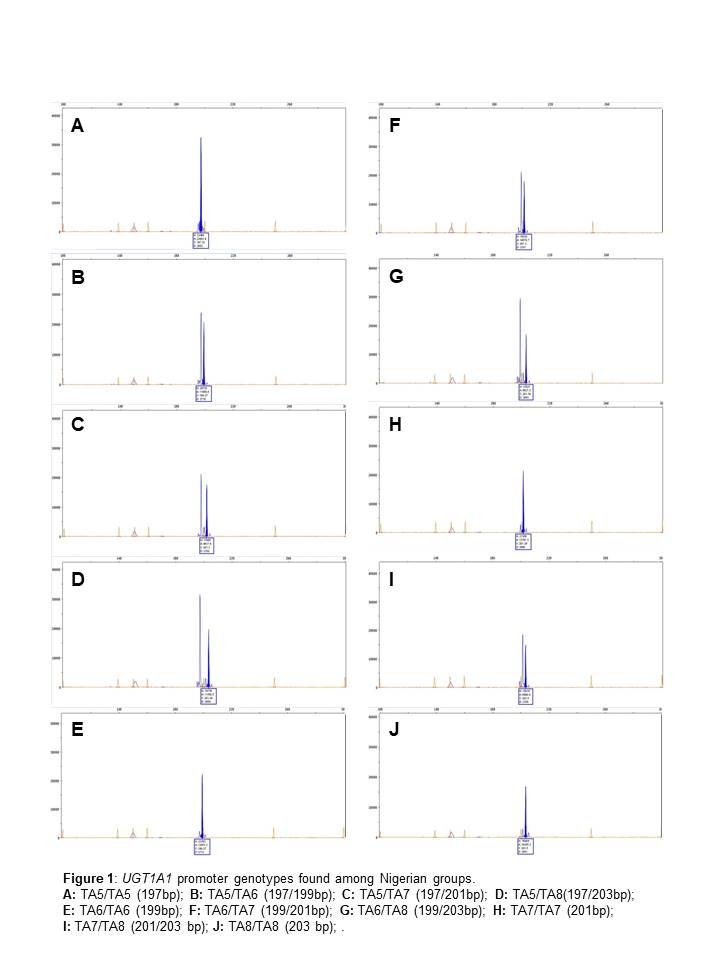
Figure S1. SUPPLEMENT MATERIAL FOR *UGT1A1* POLYMORPHISM IN YOUNG NIGERIANS WITH SCA**

Supplement: Supplementary file 1 — Additional file 1: Figure S1. Supplement material for UGT1A1 polymorphism in young Nigerians with SCA. [file 12881_2019_899_MOESM1_ESM.docx]
