# Supplementary material for: Uridine diphosphate glucuronosyl transferase 1A (UGT1A1) promoter polymorphism in young patients with sickle cell anaemia: report of the first cohort study from Nigeria
Source: BMC Med Genet. 2019 Oct 16;20:160. doi: 10.1186/s12881-019-0899-3 (PMC6794735; doi:10.1186/s12881-019-0899-3)
Supplement: Supplementary file 2 — Additional file 2: Table S1. UGT1A1 genotypes distribution with their bilirubin and LDH levels among patients. [file 12881_2019_899_MOESM2_ESM.docx]

Table S1. *UGT1A1* genotypes distribution with their bilirubin and LDH levels among patients

| *UGT1A1* Genotype | TA 5/6  N= 9 | TA5/7  N= 6 | TA5/8  N= 3 | TA6/6  N= 25 | TA6/7  N= 31 | TA6/8  N= 2 | TA/77  N= 22 | TA7/8  N= 2 | TA8/8  N= 1 |
| --- | --- | --- | --- | --- | --- | --- | --- | --- | --- |
| Total Bilirubin (mg/dl) Median (Range) | 1.4 (0.5 - 2.8) | 2.2 (1.3 – 3.2) | 1.8 (1.0 – 3.1) | 1.4 (0.4 – 3.8) | 1.8 (0.9 – 4.7) | 2.6 (1.8 – 3.4) | 2.8(1.2 – 7.8) | 5.8 (3.6 – 8.1) | 1.3(1.3 – 1.3) |
| Mean ± STD | 1.6±0.9 | 2.1±0.7 | 2.0±1.0 | 1.6±0.7 | 2.1±0.9 | 2.6±1.1 | 3.4±1.9 | 5.8±3.2 | 1.3±0 |
| Unconjugated Bilirubin (mg/dl) Median (Range) | 0.5 (0.3 - 1.6) | 1.0 (0.4 - 2.1) | 2.0 (0.6- 2.2) | 0.6 (0.1-2.8) | 0.8 (0.2-3.8) | 1.4 (0.8-1.9) | 1.7 (0.6-6.3) | 3.8 (2.0-5.6) | 0.8 (0.8-0.8) |
| Mean±STD | 0.6±0.4 | 1.0±0.6 | 1.6± 1.0 | 0.7±0.5 | 1.0 ±0.8 | 1.4±0.8 | 2.3±1.6 | 3.8±2.6 | 0.8±0 |
| LDH (IU/L) Median (Range) | 296 (197 -1300) | 458 (197 -1025) | 1350 (1040 - 1350) | 705 (296- 1417) | 789 (233- 1399) | 888 (288 - 1488) | 938 (296 - 1859) | 1133 (865 - 1400) | 1022 (1022 - 1022) |
| Mean ± STD | 462 ± 366 | 506± 312 | 1247±179 | 779± 316 | 792±318 | 888±849 | 976±420 | 1133±378 | 1022±0 |

|  |  |  |  |  |  |  |  |  |  |
| --- | --- | --- | --- | --- | --- | --- | --- | --- | --- |
|  |  |  |  |  |  |  |  |  |  |
